# Diverse histologic appearances in pulmonary mucinous cystic neoplasia: A case report

**DOI:** 10.1186/1752-1947-2-312

**Published:** 2008-09-29

**Authors:** Christine Wynveen, Behnaz Behmaram, George Haasler, Nagarjun Rao

**Affiliations:** 1 Department of Pathology, Memorial Sloane Kettering Cancer Center, New York, NY, USA; 2 Departments of Pathology Medical College of Wisconsin, Milwaukee, W. Wisconsin Avenue, WI 53226, USA; 3 Thoracic Surgery, Medical College of Wisconsin, Milwaukee, W. Wisconsin Avenue, WI 53226, USA

## Abstract

**Introduction:**

Primary pulmonary mucinous cystic neoplasia comprises a group of tumors, from benign cystadenoma to mucinous cystadenocarcinoma.

**Case presentation:**

We report a case of primary pulmonary mucinous cystadenocarcinoma in a 75-year-old woman who was found to have a right hilar mass on a routine chest X-ray. A lobectomy was performed and the resection specimen revealed a multicystic mucinous tumor. Microscopically, the tumor was composed of confluent mucin-filled cystic spaces lined by columnar mucin-secreting cells which ranged from cytologically bland to moderately atypical with 'bronchioloalveolar pattern' invasion into the adjacent parenchyma. Immunohistochemically, tumor cells were positive diffusely for Cytokeratin 7, and focally for Cytokeratin 20 and Thyroid Transcription Factor-1.

**Conclusion:**

This case highlights the continuous spectrum of pulmonary mucinous cystic neoplasia from benign mucinous cystadenoma to malignant mucinous cystadenocarcinoma, and the probable existence of a 'borderline' mucinous cystic tumor. Although molecular data are lacking to substantiate progression from benign to malignant in these neoplasms, the importance of recognizing the morphologic continuum lies in alerting pathologists to thoroughly examine specimens to rule out invasive foci in tumors with 'borderline' morphology.

## Introduction

Primary mucinous cystic tumors of the lung are rare neoplasms with a distinctly different histology from more commonly encountered pulmonary adenocarcinomas. Although these mucinous neoplasms are histologically similar to well recognized mucinous tumors of the ovary, pancreas and appendix, there has been considerable confusion in the literature with the use of various diagnostic terms, including some overlapping ones. These terms include mucinous cyst, mucinous cystadenoma, mucinous multilocular cystic carcinoma, pseudomyxomatous pulmonary adenocarcinoma, mucinous cystic tumor of low malignant potential, mucinous cystic tumor of borderline malignancy, mucinous cystadenocarcinoma, colloid carcinoma and signet ring cell carcinoma [1]. They include diverse entities that span a spectrum of histologic changes and biologic behavior ranging from benign, to borderline and malignant. Using strict diagnostic criteria, pulmonary mucinous cystic neoplasia (PMCN) may be recognized as a distinct entity [1]. Individual variation in tumor behavior occurs, that is not well predicted by histologic features. As a result, cases of 'borderline malignancy' have been reported with recurrence or metastases. Conversely, cases reported with microscopic foci of 'carcinoma' have been free of recurrence or metastases. This case illustrates the spectrum of histologic appearances in PMCN and the importance of recognizing them.

## Case presentation

**Figure 1:**
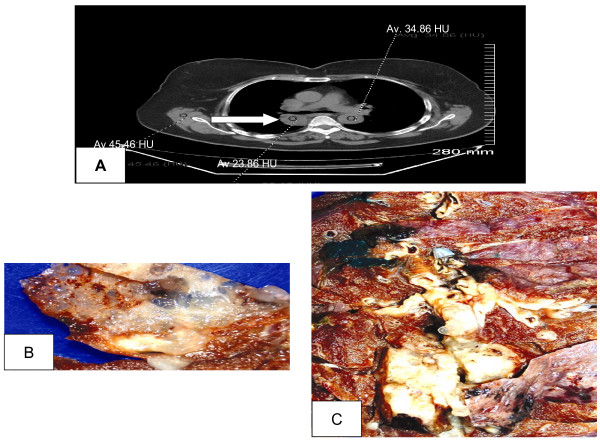
**(A) Computed tomography scan shows a well-defined mass (white arrow) with slightly lobulated margins posterior to the right hilum.** The annotations show comparative attenuations within the mass (23.86 HU), aorta (35 HU) and skeletal muscle (46 HU), indicating a cystic lesion. (B) Gross photograph demonstrating a nodular cystic mass. (C) Gross photograph demonstrating translucent cystic appearance due to the abundant mucin content.

A 75-year-old woman with a 60-year history of tobacco use was noted to have a right hilar mass on a routine chest X-ray. Computed tomography (CT) scan and magnetic resonance imaging (MRI) demonstrated a 4.5 cm well defined mass with slightly lobulated margins present between the upper and lower lobes of the right lung posterior to the right hilum (Figure 1A). The mass abutted the right main bronchus, the bronchus intermedius and the medial pleural surface. Although the visual impression of the mass radiologically suggested a solid mass, attenuation within the mass (approximately 24 Hounsfield Units (HU)) was less than in the aorta containing blood (approximately 35 HU) and skeletal muscle (approximately 46 HU) indicative of a cystic rather than a solid lesion (Figure 1A). There was no associated adenopathy or obvious compression of the airway or other structures. Positron emission tomography (PET) scan showed increased uptake in the thyroid gland which on follow-up ultrasound and fine-needle aspiration was diagnosed as multinodular colloid goiter and thyroiditis. Additional work-up was negative for metastatic disease. The patient was minimally symptomatic with a mild cough productive of yellow sputum, but with no shortness of breath or evidence of hemoptysis. The pulmonary mass was felt to represent a primary tumor and the patient underwent subsequent thoracotomy with right upper lobectomy and segmentectomy of the right lower lobe. At follow-up 2 years later, she had no evidence of recurrence or metastatic disease.

On pathological examination, the specimen consisted of resected right upper lobe of lung with attached superior segment of right lower lobe. The pleural surface showed a retracted, indurated area 4.5 × 3 cm close to the bronchus intermedius of the upper lobe. On sectioning, a 5.5 cm nodular cystic mass with a distinctly mucoid appearance (Figure 1B and 1C) was seen.

**Figure 2:**
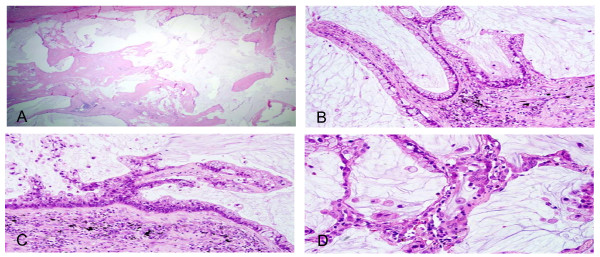
**(A) Low power microphotograph demonstrating a multicystic tumor without any distinct solid areas, partially rimmed by a fibrous capsule.** Hematoxylin and eosin, 4×. (B) Benign areas in the tumor consisting of a simple columnar mucinous epithelial lining, with frequent goblet cells. The nuclei are regular, basally oriented; without stratification, atypia or mitoses. Hematoxylin and eosin, 20×. (C) Areas of 'borderline' morphology with significant tufting, stratification and hyperchromasia of nuclei coupled with pleomorphism and mitotic activity. Hematoxylin and eosin, 20×. (D) Invasion into surrounding parenchyma in a 'lepidic/bronchioloalveolar' pattern. Mucinous tumor cells appear to line alveoli in a patchy fashion while respecting the alveolar septal framework. Hematoxylin and eosin, 40×.

Microscopically, the tumor, although situated adjacent, did not have any relationship with the bronchus, was well-demarcated from the surrounding lung, and had a multicystic appearance without any distinct solid areas (Figure 2A). The cysts contained abundant pale basophilic mucin and the cyst lining showed a spectrum of appearances. In some areas, the lining consisted of simple columnar mucinous epithelium, with frequent goblet cells. The nuclei were regular, basally oriented; without stratification, atypia or mitoses (Figure 2B). In other areas, there was significant tufting, stratification and hyperchromasia of nuclei coupled with pleomorphism and mitotic activity (Figure 2C). At the periphery of the tumor, there was evidence of invasion into surrounding parenchyma in a 'lepidic/bronchioloalveolar' pattern. Mucinous tumor cells appeared to line alveoli in a patchy fashion while respecting the alveolar septal framework (Figure 2D).

**Figure 3:**
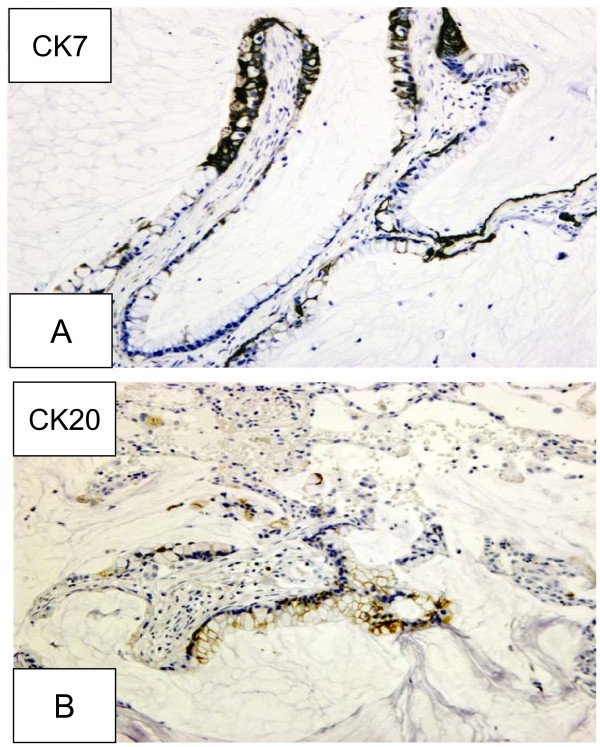
**(A) Immunohistochemistry.** CK7 positivity. (B) Immunohistochemistry. CK20 positivity.

Immunohistochemically, the tumor cells showed strong positivity for Cytokeratin 7 (CK7) (Figure 3A). Cytokeratin 20 (CK20) was focally positive (Figure 3B). Thyroid Transcription Factor-1 (TTF-1) and p53 were negative and cell proliferation marker MIB-1 showed a low positivity index (< 10%).

## Discussion

Primary mucinous cystic tumors of the lung are very rare. Following their initial description [2,3], approximately 76 cases have been reported in the English literature [1,4-10]. Clear-cut criteria for the diagnostic designation of PMCN are lacking, partly owing to the different terminologies that have been used to describe them [1]. For example, the term 'mucinous or colloid' carcinoma has been used for tumors morphologically resembling mucinous cystic tumors [4,11]. However, PMCN deserve to be a separate entity due to their unique morphology and clinical behavior that is different from other primary lung tumors. In line with this thinking, the World Health Organization (WHO) classifies mucinous cystadenocarcinoma and colloid/mucinous carcinoma separately [12]. Mucinous cystadenocarcinoma is 'a circumscribed tumor that may have a partial fibrous capsule. Centrally there is cystic change with mucin pooling and the neoplastic mucinous epithelium grows along alveolar walls'. Mucinous (colloid) carcinoma, on the other hand, is described as a 'lesion identical to their counterparts in the gastrointestinal tract, with dissecting pools of mucin containing islands of neoplastic epithelium. The epithelium in such cases may be extremely well differentiated and sometimes tumor cells float within the pools of mucin'. Mucinous cystadenoma is defined as 'a localized cystic mass filled with mucin and surrounded by a fibrous wall lined by well-differentiated columnar mucinous epithelium'.

In a recent review [1], diagnostic criteria of PMCN have been proposed based on well-defined morphologic features including: 1) solitary, well-circumscribed tumors with a cystic gross appearance; 2) no evidence of carcinoma *in situ *in the bronchial mucosa; 3) mucin comprising greater than 90% of the tumor bulk; 4) neoplastic epithelial cells are mucin-producing and are found lining the fibrous wall or floating within mucin; and 5) no evidence of another primary mucinous malignant neoplasm. In this analysis, the presence of solid invasive areas and marked cytologic atypia, including prominent nuclear stratification, nuclear enlargement or high nuclear/cytoplasmic ratio, prominent nucleoli, and frequent mitoses, indicated malignancy. However, histologic features may not be very accurate in predicting progression. Three separate reports comprising five cases of mucinous cystadenocarcinoma demonstrated no evidence of recurrence or metastasis after a range of 1.5 to 6 years of follow-up [6-8]. Also, in a series of 11 cases of 'borderline' mucinous cystic tumors, all patients were alive and well after a mean follow-up of 4.7 years, despite the presence of microscopic foci of carcinoma in four cases [5]. Conversely, distant metastasis has been reported in a case of a 'multilocular mucous cyst' in which seeding of the parietal pleura was noted at the time of surgery [3]. Additionally, a case of local recurrence of a borderline mucinous cystic tumor after limited resection has also been reported [13], as has been a patient with late recurrence of mucinous cystadenoma [14].

The variation in biologic behavior, especially in the 'borderline' category, brings into question the necessity of having such a category for diagnostic purposes. In fact, the WHO classification does not recognize pulmonary borderline mucinous cystic tumor/mucinous cystic tumor with atypia as a distinct entity [12]. However, the borderline category still has its uses as outlined below. First, recognition of borderline features within a tumor ensures adequate sampling of the lesion by pathologists so that histologically malignant foci are not missed. Secondly, clinicians are alerted to the need for close follow-up in such cases. Therefore, until molecular genetic lesions underlying this group of tumors are elucidated, borderline mucinous tumor should not be eliminated as a diagnostic category. In the current case, the existence of the full spectrum of benign, borderline and malignant histology within a single tumor suggests that these tumors may be analogous to type I ovarian tumors according to the model proposed by Shih and Kurman [15].

The differential diagnosis of PMCN includes mucous gland adenoma, mucoepidermoid carcinoma, mucinous bronchioloalveolar carcinoma and metastatic mucinous carcinoma. Mucous gland adenoma and mucoepidermoid carcinoma are both endobronchial lesions in contrast to the peripheral location of PMCN. Mucous gland adenoma exhibits cystically dilated mucin glands protruding into the bronchial lumen while mucoepidermoid carcinoma is composed of mucin-producing cells admixed with squamous and intermediate cells [12]. A thorough clinical and radiologic evaluation is necessary to exclude a metastasis of mucinous carcinoma from another organ. The immunohistochemical profile in the current case consisted of strong diffuse positivity for CK7 and focal positivity for CK20, whereas TTF-1 was negative. Such an immunoprofile may be a source for diagnostic confusion. It is important to remember that CK20 positivity has been described in some types of primary pulmonary mucinous lesions [8]. Differentiating pulmonary mucinous cystadenocarcinoma from mucinous bronchioloalveolar carcinoma may pose a challenge, especially in those unusual cases where necrosis leads to secondary cavitation or cyst formation in the latter. Necrosis is conspicuously absent in mucinous cystadenocarcinoma. Growth pattern is another differentiating feature. While PMCN are solitary, generally well circumscribed tumors, mucinous bronchioloalveolar carcinoma is diffuse and often bilateral, appearing grossly as an ill-defined lobar consolidation.

The optimal management of PMCN appears to be complete surgical excision by lobectomy. The role of adjuvant therapy is unclear, but likely limited. In our patient, the tumor was resected with clear margins and all 15 lymph nodes submitted for histological examination were negative for metastasis. Although our patient's prognosis is favorable, close long-term follow-up is clearly indicated.

## Conclusion

In summary, we have reported a case of pulmonary mucinous cystadenocarcinoma which showed histologic changes extending from the benign to the malignant end of the spectrum. The pathologist's role in such cases lies in recognizing the entity, distinguishing it from other mucinous lesions in the lung and alerting the clinician to the probability of aggressive behavior in the presence of atypical 'borderline' histology and invasive foci.

## Competing interests

The authors declare that they have no competing interests.

## Authors' contributions

CW made substantial contributions to acquisition, analysis and interpretation of data, was involved in drafting the manuscript and has given final approval of the version to be published. BB made substantial contributions to conception and design, was involved in revising the manuscript critically for important intellectual content, and has given final approval of the version to be published. GH made substantial contributions to acquisition, analysis and interpretation of data, was involved in drafting the manuscript and revising it critically for important intellectual content, and has given final approval of the version to be published. NR made substantial contributions to conception and design, acquisition, analysis and interpretation of data, was involved in drafting the manuscript and revising it critically for important intellectual content, and has given final approval of the version to be published.

## Consent

Written informed consent was obtained from the patient for publication of this case report and any accompanying images. A copy of the written consent is available for review by the Editor-in-Chief of this journal.
